# The Malaysian Medication Adherence Scale (MALMAS): Concurrent Validity Using a Clinical Measure among People with Type 2 Diabetes in Malaysia

**DOI:** 10.1371/journal.pone.0124275

**Published:** 2015-04-24

**Authors:** Wen Wei Chung, Siew Siang Chua, Pauline Siew Mei Lai, Donald E. Morisky

**Affiliations:** 1 Department of Pharmacy, Faculty of Medicine, University of Malaya, Kuala Lumpur, Malaysia; 2 Pharmacy Department, University Malaya Medical Centre, Kuala Lumpur, Malaysia; 3 Department of Primary Care Medicine, University Malaya Primary Care Research Group (UMPCRG), Faculty of Medicine, University of Malaya, Kuala Lumpur, Malaysia; 4 Department of Community Health Sciences, UCLA Fielding School of Public Health, Los Angeles, California, United States of America; Carl von Ossietzky University of Oldenburg, GERMANY

## Abstract

Medication non-adherence is a prevalent problem worldwide but up to today, no gold standard is available to assess such behavior. This study was to evaluate the psychometric properties, particularly the concurrent validity of the English version of the Malaysian Medication Adherence Scale (MALMAS) among people with type 2 diabetes in Malaysia. Individuals with type 2 diabetes, aged 21 years and above, using at least one anti-diabetes agent and could communicate in English were recruited. The MALMAS was compared with the 8-item Morisky Medication Adherence Scale (MMAS-8) to assess its convergent validity while concurrent validity was evaluated based on the levels of glycated hemoglobin (HbA1C). Participants answered the MALMAS twice: at baseline and 4 weeks later. The study involved 136 participants. The MALMAS achieved acceptable internal consistency (Cronbach’s alpha=0.565) and stable reliability as the test-retest scores showed fair correlation (Spearman’s rho=0.412). The MALMAS has good correlation with the MMAS-8 (Spearman’s rho=0.715). Participants who were adherent to their anti-diabetes medications had significantly lower median HbA1C values than those who were non-adherence (7.90 versus 8.55%, p=0.032). The odds of participants who were adherent to their medications achieving good glycemic control was 3.36 times (95% confidence interval: 1.09-10.37) of those who were non-adherence. This confirms the concurrent validity of the MALMAS. The sensitivity of the MALMAS was 88.9% while its specificity was 29.6%. The findings of this study further substantiates the reliability and validity of the MALMAS, in particular its concurrent validity and sensitivity for assessing medication adherence of people with type 2 diabetes in Malaysia.

## Introduction

The World Health Organization (WHO) defined medication adherence as “the extent to which a person’s behavior in terms of taking medications, following diets or executing lifestyle changes, corresponds with agreed recommendations from the healthcare provider” [1]. Although a variety of treatment options is available, only one third of people with chronic diseases adhered to their medication regimens [2]. Medication non-adherence is a prevalent concern in translating effective treatment in research settings to clinical practice [3].

Depending on the complexity of the prescribed medication regimens, generally 40% of patients do not adhere to their medications [4–7]. When complex medication regimens were coupled with other requirements such as lifestyle changes, medication non-adherence was estimated to reach as high as 70% [2,5–9].

Studies found that adherence to oral anti-diabetes agents varied between 30% and 98%. This wide variation may be attributed to the different definitions used, duration of study, outcome measures and assessment methods, types of treatment and number of medications used [10–11]. In developed countries such as the United States (US), it has been estimated that 30% to 50% of patients on medications for chronic diseases are non-adherent to their medication therapies [1,12]. Medication non-adherence was higher in developing countries such as Gambia (73%) and China (54%) [1].

Medication non-adherence is also a costly economic burden worldwide [3,12–14] with an estimated annual disease-related medical cost of USD100 billion [15–18]. Besides limiting the effectiveness of treatment and increasing healthcare costs [19–22], medication non-adherence also leads to a rise in morbidity and mortality rates [23].

Potential determinants of medication adherence could be divided into factors related to patient, medication and the healthcare system [11]. These include demographic and behavioral characteristics of patients, patients’ knowledge and awareness concerning their medications and diseases, the complex nature, convenience and cost of treatment as well as the occurrence of side effects [16–18,20,24–28].

According to the WHO, “Poor adherence to long-term therapies severely compromises the effectiveness of treatment making this a critical issue in population health both from the perspective of quality of life and of health economics” [1]. For instance, Wood found that people with diabetes who were adherent to their medication regimens were 48% less likely to die prematurely [29].

A qualitative study conducted by Manias deduced that in general, patients perceived the clinician as a prescriber and one who manages their chronic conditions. The pharmacist, on the other hand is perceived as a central educator to assist patients in managing their chronic conditions and medication regimens as well as any issues related to adverse effects and medication adherence [30]. Nonetheless, the responsibility to enhance medication adherence should reside on all healthcare professionals and the patients.

Although many studies had been conducted to improve medication adherence, currently there is no gold standard available to assess this issue [1,15]. Various methods which have been used to assess medication adherence can be divided into direct methods (such as monitoring of serum drug levels and direct observation of therapy), and indirect methods (such as patient self-report, pill counts, rate of prescription refills, patient diaries and electronic container monitors) [3,31]. Each method of assessment has its advantages and disadvantages. For example, the self-reporting method suffers from low specificity (50%) but has high sensitivity (90%) [3].

The Morisky, Green and Levine Scale has been commonly used for the assessment of medication adherence [29,31–33]. This instrument was originally developed with 4 items only [33–34] but has since been modified to the current 8-item Morisky Medication Adherence Scale, MMAS-8 [27,35]. The MMAS-8 is supposed to be an integral scale for assessing general medication adherence behavior and hence, does not specify the duration of recall.

Lu et al demonstrated that self-reported medication adherence was dependent on the recall period and a one-month duration was deemed practical [36]. Therefore, the Malaysian Medication Adherence Scale (MALMAS) was developed based on the hypothesis that patients could comprehend their medication adherence behavior more easily if a recall period was specified. Therefore, the MMAS-8 is for assessing the general behavior of a patient while the MALMAS can be used when a specific recall period of one month is required.

Although the MALMAS has undergone a validation process previously [31], several limitations was observed and these have been addressed in the current study. Therefore, the aim of this study was to further evaluate and substantiate the psychometric properties of the English version of MALMAS, particularly its concurrent validity among people with type 2 diabetes in Malaysia.

## Patients and Methods

The MALMAS was developed based on the MMAS with 9 items (MMAS-9) [37]. One of the items in MMAS-9 about the use of a reminder system was omitted as this item measures a determinant of adherence and not the behavior itself. The other 8 items were modified to make them easier to understand and hence, the MALMAS has eight items with one domain [31].

The first item of the MALMAS has five responses: (1) All the time, (2) Often, (3) Sometimes, (4) Rarely and (5) Never. The remaining seven items have a dichotomous response of “Yes” or “No”. MMAS-9 has not been validated and hence, the MALMAS was compared with the validated MMAS-8 to assess its convergent validity [27]. The responses in the MALMAS were scored based on the MMAS-8 [27] where the total score ranged from 0 to 8. Both instruments categorized medication adherence based on the total scores obtained: low adherence (total score<6), medium adherence (6 to < 8) and high adherence (total score = 8) [27,35].

A research team consisting of 12 experienced pharmacists developed and established the face and content validity of the MALMAS. In addition, a pilot study was conducted on five patients to obtain their feedback on the clarity of the MALMAS [31].

People with type 2 diabetes, aged 21 years and above, who were using at least one anti-diabetes agent and could communicate in English, were recruited from the diabetes clinic of a major teaching hospital in Malaysia from April to May 2013. Based on convenience sampling, any person who met the inclusion criteria and visited the diabetes clinic during the study period would be approached to participate in the study while he/she was waiting to see the doctor. A researcher explained the purpose and study procedure to potential participants and requested them to complete a set of questionnaires, which consisted of the MALMAS and the MMAS-8. The participants took approximately 5 to 10 minutes to complete the questionnaires.

The researcher counter-checked the completed questionnaire to ensure that all the questions were answered. The same participants answered the questionnaire again four weeks later. The Medical Ethics Committee of the University Malaya Medical Centre approved the study in writing (MEC Ref. No. 757.112). The study was conducted according to the principles expressed in the Declaration of Helsinki. All participants provided their written, informed consent upon recruitment into this study.

## Data Analysis

All data were analyzed using the PASW Statistics for Windows, Version 18 (SPSS Inc., Chicago). Using the Kolmogorov-Smirnov test, data obtained from both the MALMAS and the MMAS-8 did not fulfill the normal distribution criteria. Any possible associations between categorical variables were analyzed using chi-square (χ^2^) test, while Wilcoxon signed ranks test or Mann-Whitney U test was used for testing the difference in continuous variables between two groups.

Cronbach’s alpha values were used to determine the internal consistency of the MALMAS and the MMAS-8. A Cronbach’s alpha value of more than 0.5 is considered as acceptable [38–40]. When an item is deleted from a scale and leads to a significant increase in Cronbach’s alpha, then this item should be excluded to produce a more homogeneous scale [38]. On the other hand, the corrected item-total correlations indicate how much each item in an instrument correlates to its total score. These should be higher than 0.2 to be acceptable [38]. Any item with a value less than 0.2 means that it is assessing a different construct from that of the whole instrument [38,41].

To determine whether the MALMAS has stable reliability, the test-retest results were compared using McNemar test, Wilcoxon Signed Ranks test and Spearman’s rho. Spearman’s rho is interpreted as followed: little or no correlation (0–0.25), fair correlation (0.25–0.5), moderate to good correlation (0.5–0.75) and very good to excellent correlation (> 0.75) [42].

Concurrent validity was assessed by analyzing the levels of medication adherence and participants’ HbA_1c_ values, using chi-square test, Kruskal-Wallis H test and Mann-Whitney U test. The correlation between the total score of the MALMAS and the HbA_1c_ values of the participants was determined using Spearman’s rank order correlation coefficient.

The sensitivity and specificity as well as the positive and negative predictive values of the MALMAS were generated. The sensitivity of the MALMAS determines its ability to correctly predict poor glycemic control in patients who are non-adherent to their medications, while the specificity of the MALMAS determines its ability to correctly predict good glycemic control in patients who are adherent to their medications [43]. Positive predictive value (also known as precision rate) is the proportion of positive test results that are true positives [43]. This value quantifies how likely participants with poor glycemic control are also non-adherent to their medications, and vice versa. On the other hand, the negative predictive value is the proportion of participants with a negative test result [43]. This value quantifies how likely participants with good glycemic control are also adherent to their medications.

## Results

A total of 136 participants were recruited in this study and their demographic and clinical data are presented in Table 1.

**Table 1:** Demographic and clinical data of participants according to the level of medication adherence (N = 136).

Characteristics	Total sample	Low adherence	Medium adherence	High adherence
	N (%)	n (%)	n (%)	n (%)
**Age in years**				
Mean ± SD	58.1 ± 10.2	53.2 ± 9.3	58.2 ± 10.3	62.6 ± 9.0
(Median) [Range]	(57.0) [32–83]	(54.0) [35–74]	(57.5) [32–78]	(63.0) [43–83]
**Gender**				
Male	63 (46.3)	16 (44.4)	28 (47.5)	19 (46.3)
Female	73 (53.7)	20 (55.6)	31 (52.5)	22 (53.7)
**Ethnic group**				
Malay	54 (39.7)	17 (47.2)	25 (42.4)	12 (29.3)
Chinese	28 (20.6)	3 (8.3)	14 (23.7)	11 (26.8)
Indian	50 (36.8)	16 (44.4)	18 (30.5)	16 (39.0)
Others	4 (2.9)	0 (0)	2 (3.4)	2 (4.9)
**Working status**				
Working	48 (35.3)	21 (58.3)	21 (35.6)	6 (14.6)
Not working	88 (64.7)	15 (41.7)	38 (64.4)	35 (85.4)
**Education level**				
None or primary	14 (10.4)	3 (8.1)	7 (11.9)	4 (10.0)
Secondary	71 (52.2)	19 (51.4)	29 (49.2)	23 (57.5)
Diploma	21 (15.4)	6 (16.2)	10 (16.9)	5 (12.5)
Tertiary/postgraduate	30 (22.0)	9 (24.3)	13 (22.0)	8 (20.0)
**Duration of diabetes in years**				
Mean ± SD	15.6 ± 8.3	14.0 ± 7.1	14.9 ± 7.6	17.8 ± 10.0
(Median)][Range]	(15.0) [1–43]	(13.0) [1–33]	(13.5) [3–35]	(17.0) [1–43]
**No. of prescribed medications**				
Mean ± SD	7.5 ± 3.0	7.3 ± 2.4	7.4 ± 3.6	8.0 ± 2.3
(Median)][Range]	(7.0) [3–19]	(7.0) [3–14]	(7.0) [3–19]	(8.0) [3–12]
**No. of oral hypoglycemic agents**				
Mean ± SD	1.4 ± 0.9	1.4 ± 0.9	1.5 ± 0.9	1.3 ± 1.0
(Median)][Range]	(1.0) [0–4]	(1.0) [0–3]	(1.0)[0–4]	(1.0) [0–3]
**No. of insulin**				
Mean ± SD	1.3 ± 0.9	1.4 ± 0.8	1.3 ± 0.9	1.4 ± 0.9
(Median)][Range]	(2.0) [0–3]	(2.0) [0–3]	(2.0) [0–2]	(2.0) [0–3]
**FBG (mmol/L)**				
Mean ± SD	8.8 ± 4.1	9.5 ± 4.0	9.0 ± 4.5	7.7 ± 3.1
(Median) [Range]	(7.7) [2.8–29.6]	(8.4) [4.1–20.0]	(7.7) [2.8–29.6]	(7.0) [3.8–19.0]
**HbA**_**1c**_ **(%)**				
Mean ± SD	8.5 ± 2.0	8.8 ± 1.7	8.6 ± 2.2	7.9 ± 1.9
(Median) [Range]	(8.1) [5.2–15.3]	(8.6) [6.4–13.0]	(8.1) [5.2–15.3]	(7.6) [5.7–15.1]

SD, standard deviation; FBG, fasting blood glucose

### Psychometric properties of the MALMAS

#### Reliability Analysis

The MALMAS and MMAS-8 produced Cronbach’s alpha values of 0.565 and 0.595, respectively. However, the Cronbach’s alpha of MALMAS would increase to 0.571 if item 3 was excluded; while for MMAS-8, this value would increase to 0.598 if item 6 was deleted (Table 2). The item-total correlations were less than 0.2 for three items in the MALMAS (items 3, 4 and 5) and two items in the MMAS-8 (items 5 and 6) [Table 2].

**Table 2:** Reliability analysis of the MALMAS and the MMAS-8.

Item number	Corrected item total correlations		Cronbach’s alpha if item deleted		Test-retest reliability McNemar test / Wilcoxon signed ranks test	
					p-value	p-value
	MALMAS	MMAS-8	MALMAS	MMAS-8	MALMAS	MMAS-8
**1 for MALMAS and 8 for MMAS-8**	0.515	0.534	0.485	0.530	0.022*	0.001**
					(-2.292^c^)	(-3.477^c^)
**2**	0.423	0.478	0.469	0.491	0.035*	0.136
**3**	0.185^a^	0.258	0.571^b^	0.576	0.871	0.011*
**4**	0.181^a^	0.202	0.562	0.586	0.383	1.000
**5**	0.159^a^	0.140^a^	0.561	0.597	0.180	0.219
**6**	0.227	0.133^a^	0.570	0.598^b^	1.000	1.000
**7**	0.251	0.288	0.539	0.567	0.503	0.720
**8 for MALMAS and 1 for MMAS-8**	0.382	0.429	0.531	0.515	0.286	0.032*
Total score					<0.001**	<0.001**
(Spearman’s correlation coefficient)					(0.412^d^)	(0.376^d^)

NA, Not applicable;

*p < 0.05;

**p<0.001

^a^Corrected item-total correlations < 0.2;

^b^Increase in Cronbach’s alpha value if item was deleted;

^c^z value obtained from Wilcoxon Signed Ranks test;

^d^Spearman’s rho

The Spearman’s rho (ρ) for the test-retest results were 0.412 (p<0.001) for MALMAS and 0.376 (p<0.001) for MMAS-8. When the individual items in the MALMAS were analyzed, two out of the 8 items were significantly different at test retest while for the MMAS-8, three items were significantly different.

#### Convergent Validity

The results obtained with the MALMAS and that with MMAS-8 showed no significant difference (Table 3). A moderate to good correlation was obtained between the MALMAS and MMAS-8 (Spearman’s rho = 0.715, p<0.001).

**Table 3:** Comparison between the MALMAS and the MMAS-8.

Adherence status	MALMAS	MMAS-8	χ^2^ / z value	p-value
(N = 136)	n (%)	n (%)		
**High adherence (scores = 8)**	41 (30.1)	49 (36.0)	-0.847	0.397
**Medium adherence(6 to < 8)**	59 (43.4)	49 (36.0)		
**Low adherence (0 to < 6)**	36 (26.5)	38 (27.9)		
**High adherence (scores = 8)**	41 (30.1)	50 (36.8)	-1.800	0.072
**Medium & low adherence (scores <8)**	95 (69.9)	86 (63.2)		
**High & medium adherence (scores = 6–8)**	100 (73.5)	98 (72.1)	-0.655	0.513
**Low adherence (scores <6)**	36 (26.5)	38 (27.9)		
**Mean total scores ± SD**	6.7 **±** 1.3	6.6 **±** 1.4	1.038^a^	0.299
**[Median]**	[7.0]	[7.0]		

SD, standard deviation

^a^z value was obtained using the Wilcoxon Signed Ranks Test

#### Concurrent Validity

The median values of HbA_1c_ were significantly different between the levels of medication adherence, irrespective of how these levels were grouped together (Table 4). However, significant association with glycemic control (based on HbA_1c_ values) was only observed when medication adherence was classified into two categories: high or medium adherence (considered as the adherence group) and low adherence (considered as the non-adherence group). The odds of a person with medium or high adherence achieving glycemic control is 3.36 (95% CI = 1.09–10.37) times more than those with low adherence (Table 4). In addition, a weak but significant inverse correlation was found between HbA_1c_ values and the total scores of MALMAS (Spearman’s rho = -0.212, p = 0.014).

**Table 4:** Association between medication adherence using the MALMAS and glycemic control.

Adherence	HbA_1c_	Mean rank	z value/ H^2^	Good control	Poor control	χ^2^	Odds ratio
(N = 134)	Mean + SD [Median]		(p value)	HbA_1c_ < 7%	HbA_1c_ > 7%	(p value)	(95% CI)
				n (%)	n (%)		
**High adherence**	8.00 ± 1.87 [7.60]	57.51	6.073^a^	13 (39.4)	28 (27.7)	5.01	-
**Medium adherence**	8.48 ± 2.08 [8.10]	67.20	(0.048*)	16 (48,5)	41 (40.6)	(0.081)	
**Low adherence**	8.98 ± 1.86 [8.55]	79.35		4 (12.1)	32 (31.7)		
**Medium and high adherence**	8.28 ± 2.00 [7.90]	63.15	-2.142^b^	29 (87.9)	69 (68.3)	4.84	3.362
**Low adherence**	8.97 ± 1.86 [8.55]	79.35	(0.032^c^)	4 (12.1)	32 (31.7)	(0.028*)	(1.090–10.370)
**High adherence**	8.00 ± 1.87 [7.60]	57.51	-1.978^b^	13 (39.4)	28 (27.7)	1.59	1.695
**Low and medium adherence**	8.67 ± 2.00 [8.40]	71.90	(0.048^c^)	20 (60.6)	73 (72.3)	(0.207)	(0.744–3.859)

* p < 0.05; SD, standard deviation; CI, confidence interval

^a^ H^2^ value—Kruskal-Wallis H Test; was performed when there was more than 2 groups of adherence levels;

^b^ z value—Mann-Whitney U Test; was performed when there was 2 groups of adherence levels;

#### Sensitivity and Specificity

The association between medication adherence and glycemic control is shown in Table 5. Thirty-two out of 36 participants (88.9%) showed true positive values. This means that they were non-adherent to their medications and had poor glycemic control. However, 69 out of 98 participants (70.4%) had false positive results, indicating that they were adherent to their medications but had poor glycemic control. On the other hand, 29 out of 98 participants (29.6%) had true negative results, which meant that they were adherent to their medications and had good glycemic control, while 4 out of 36 participants (11.1%) had false negative results, which meant that they were non-adherent to their medications but had good glycemic control.

**Table 5:** Sensitivity and specificity of the MALMAS.

Clinical data	Low adherence	Medium and high adherence	Positive and negative predictive value
(N = 134)	n (%)	n (%)	
			**Positive PV**
**Poor control**	32 (88.9) [TP]	69 (70.4) [FP]	TP / (TP + FP)x100%
**HbA**_**1c**_ **> 7%**			31.7%
			**Negative PV**
**Good control**	4 (11.1) [FN]	29 (29.6) [TN]	TN / (TN + FN) x100%
**HbA**_**1c**_ **< 7%**			87.9%
**Sensitivity and specificity**	**Sensitivity**	**Specificity**	
	TP / (TP + FN) x100%	TN / (TN +FP) x100%	
	88.9%	29.6%	

PV, Predictive value; TP, True positive; TN, True negative; FP, False positive; FN, False negative

The sensitivity and specificity of the MALMAS was 88.9% and 29.6%, respectively. The positive and negative predictive values were calculated as 31.7% and 87.9%, respectively.

## Discussion

This study further established the psychometric properties of the MALMAS in terms of its acceptable internal consistency, stable reliability, concurrent validity and high sensitivity. This implies that the MALMAS is a reliable and valid instrument for assessing adherence to medications.

The Cronbach’s alpha of the MALMAS was more than 0.5, which indicates that the MALMAS has acceptable internal consistency [38]. The Cronbach’s alpha of the MALMAS would increase slightly if item 3 was excluded. However, this item was retained as there were only 8 items in this instrument, and also the deletion of item 3 only increased the Cronbach’s alpha marginally. The findings from this study concurred with that of a previous validation study on the MALMAS [31].

Six out of eight items in the MALMAS were not statistically different at test-retest. In addition, Spearman’s rho for the overall scores of the MALMAS showed fair correlation between the test-retest. This indicates that the MALMAS has achieved stable reliability.

The prevalence of medication non-adherence determined using the MALMAS was similar to that of the MMAS-8, confirming the convergent validity of the MALMAS. In addition, these two instruments showed moderate to good correlation. These are similar to that of previous studies that assessed medication adherence using the MMAS-8 [44–45]. Both the MALMAS and the MMAS-8 can be used to assess adherence to long-term medications. In addition, the MMAS-8 can be used for assessing short-term medications (less than a month) but not the MALMAS since a specific recall period of a month is required. However, the MALMAS is a more appropriate instrument for comparison of medication adherence between different studies than the MMAS-8, as the recall period is not specified in the latter.

The weak inverse correlation between HbA_1c_ values and the total MALMAS scores of the participants indicates that it is better to categorize the total MALMAS scores into two groups: adherence and non-adherence, rather than using the actual score to assess medication adherence. The odds of participants who were adherent to their anti-diabetes medications and achieving good glycemic control (HbA_1c_ less than 7%) was 3.36 times of those who were non-adherence.

Participants who were non-adherence also had significantly higher median HbA_1c_ levels than those who were adherence. This confirms the concurrent validity of the MALMAS and the findings of previous studies where improving medication adherence would lead to better glycemic control in people with diabetes [46–47]. Previous studies have shown that a 10% improvement in medication adherence is associated with a decrease in HbA_1c_ by 0.16% [46–47]. Therefore, efforts to improve glycemic outcomes should include an emphasis on medication adherence, which can be assessed using the MALMAS.

The MALMAS would be considered as an accurate instrument if a high number of true positives and true negatives were obtained, when compared to false positives and false negatives [43]. The sensitivity of the MALMAS in identifying people who were non-adherent to their medications and had poor glycemic control was 88.9%. However, the specificity of the MALMAS in accurately determining participants who were adherent and also had good glycemic control was only 29.6%; indicating that the MALMAS is sensitive in assessing non-adherence to medication; but lacks specificity. These findings are similar to previous studies which used the MMAS-8 [3,27].

The high sensitivity and low specificity of the MALMAS is probably due to the small sample size [48,49] or under-reporting of non-adherence by participants [42]. Studies have shown that patients tend to be more truthful in reporting their non-adherence to medications when they are not worried about criticism [4,50]. This is possible through continuity of care where the researcher could develop a good rapport with the participants.

One of the limitations of the present study was that the MALMAS was validated only in English. Thus, only patients who could communicate in English were recruited. However, Malaysia is a multiracial society, where other languages such as Malay, Mandarin and Tamil are also widely used. Therefore, there is a need to translate and validate the MALMAS in these languages so that studies on medication adherence in Malaysia can include participants from different ethnic groups, which will better represent its multiethnic population.

## Conclusions

The concurrent validity of the MALMAS shows significant association between medication adherence and glycemic control while the other psychometric properties of the MALMAS conformed to that of other studies. In addition, the MALMAS has high sensitivity for identifying those who were non-adherent to their medications and had poor glycemic control. Therefore, this study further substantiates the reliability and validity, in particular the concurrent validity and sensitivity of the MALMAS for assessing adherence to long-term medications where a recall period of a month is required.
